# Supplementary material for: The effectiveness of a multi-domain electronic feedback report on the performance of quality indicators for chronic conditions: Protocol for a randomized controlled trial in general practice
Source: PLoS One. 2024 Nov 21;19(11):e0314360. doi: 10.1371/journal.pone.0314360 (PMC11581287; doi:10.1371/journal.pone.0314360)
Supplement: S2 Appendix — (PDF) [file pone.0314360.s002.pdf]

## Operationalized criteria for the identification of chronic conditions and for the quality indicator definitions in the FIRE database

| Condition | ICD-10 codes | ICPC-2 codes | Clinical parameters | Laboratory parameters | Medication |
|---|---|---|---|---|---|
| Asthma | J45 (Asthma) | R96 (Asthma) | - | - | - |
| Atrial fibrillation or flutter | I48 (Atrial fibrillation and flutter) | K78 (Atrial fibrillation/flutter) | - | - | - |
| Coronary heart disease | I20-25 (Ischemic heart disease) | K74 (Ischemic heart disease w. angina), K75 (Acute myocardial infarction), K76 (Ischemic heart disease w/o angina) | - | - | ATC code in group C01D (Vasodilators used in cardiac diseases) |
| Chronic kidney disease | N18 (Chronic kidney disease) | - | - | At least two estimated glomerular filtration rate values <60 ml/min/1.73m$^2$ over 90 days apart [1] | - |
| Chronic obstructive pulmonary disease | J44 (Other chronic obstructive pulmonary disease) | R95 (Chronic obstructive pulmonary disease) | - | - | - |
| Diabetes mellitus | E10-14 (Diabetes mellitus) | T89 (Diabetes insulin dependent), T90 (Diabetes non-insulin dependent) | - | At least two consecutive glycated hemoglobin values ≥6.5 mmol/L [2] | ATC code in group A10 (Drugs used in diabetes) except A10BJ (Glucagon-like peptide-1 analogues) |
| Hypertension | I10-15 (Hypertensive diseases) | K85 (Elevated blood pressure), K86 (Hypertension uncomplicated), K87 (Hypertension complicated) | At least one of the following [3]:<br>– At least two blood pressure readings ≥140 mmHg systolic and/or ≥90 mmHg diastolic between 7 and 180 days apart<br>– At least one blood pressure reading ≥180 mmHg systolic and/or ≥110 mmHg diastolic | - | Global Trade Item Number in the Pharmaceutical Cost Group “Hypertension” (hypertension) [4] |

Abbreviations: ATC, Anatomic Therapeutic Chemical classification system; ICD-10, International Classification of Disease; ICPC-2, International Classification of Primary Care.

| No. | Domain | Quality indicator description | Criteria for quality indicator eligibility (denominator population) | Criteria for quality indicator achievement (numerator population) |
|---|---|---|---|---|
| 1 | Cardiovascular | Proportion of patients with hypertension aged <80 years whose latest BP measurement during the past 12 months was <140/90 mmHg (primary treatment target) or who were prescribed at least three antihypertensive drugs of different classes during the past 12 months. | Hypertension and age <80 years | At least one of the following during the past 12 months:<br>– Latest BP <140 mmHg systolic and <90 mmHg diastolic<br>– Among medications in the pharmacological cost group “Hypertonie” (hypertension) [4], at least one ATC code from three different of the following categories:<br>– Diuretics: C03AA03 (Hydrochlorothiazide), C03BA11 (Indapamide), C03EA01 (Hydrochlorothiazide and potassium-sparing agents), C07BB07 (Clofenamide and potassium), C07CB03 (Atenolol and other diuretics), C09BA (ACE inhibitors and diuretics), C09BX01 (Perindopril, amlodipine and indapamide), C09DA (Angiotensin II receptor blockers (ARBs) and diuretics), C09DX01 (Valsartan, amlodipine and hydrochlorothiazide), C09DX03 (Olmesartan medoxomil, amlodipine and hydrochlorothiazide), C09XA52 (Aliskiren and hydrochlorothiazide)<br>– Beta blocking agents: C07AA05 (Propranolol), C07AB02 (Metoprolol), C07AB03 (Atenolol), C07AB07 (Bisoprolol), C07AB08 (Celiprolol), C07AB12 (Nebivolol), C07AG01 (Labetalol), C07AG02 (Carvedilol), C07BB07 (Bisoprolol and thiazides), C07CB03 (Atenolol and other diuretics), C09BX02 (Perindopril and bisoprolol)<br>– Calcium channel blockers: C08CA01 (Amlodipine), C08CA02 (Felodipine), C08CA03 (Isradipine), C08CA05 (Nifedipine), C08CA13 (Lercanidipine), C08DA01 (Verapamil), C08DB01 (Diltiazem), C09BB02 (Enalapril and lercanidipine), C09BB04 (Perindopril and amlodipine), C09BB10 (Trandolapril and verapamil), C09BX01 (Perindopril, amlodipine and indapamide), C09DB01 (Valsartan and amlodipine), C09DB02 (Olmesartan medoxomil and amlodipine), C09DB07 (Candesartan and amlodipine), C09DX01 (Valsartan, amlodipine and hydrochlorothiazide), C09DX03 (Olmesartan medoxomil, amlodipine, and hydrochlorothiazide) |

| No. | Domain | Quality indicator description | Criteria for quality indicator eligibility (denominator population) | Criteria for quality indicator achievement (numerator population) |
|---|---|---|---|---|
| | | | | – Angiotensin-converting enzyme inhibitors: C09AA01 (Captopril), C09AA02 (Enalapril), C09AA03 (Lisinopril), C09AA04 (Perindopril), C09AA05 (Ramipril), C09AA06 (Quinapril), C09AA07 (Benazepril), C09BA (ACE inhibitors and diuretics), C09BB02 (Enalapril and lercanidipine), C09BB04 (Perindopril and amlodipine), C09BB10 (Trandolapril and verapamil), C09BX01 (Perindopril, amlodipine and indapamide), C09BX02 (Perindopril and bisoprolol)<br>– Angiotensin II receptor blockers: C09CA01 (Losartan), C09CA02 (Eprosartan), C09CA03 (Valsartan), C09CA04 (Irbesartan), C09CA06 (Candesartan), C09CA07 (Telmisartan), C09CA08 (Olmesartan medoxomil), C09CA09 (Azilsartan medoxomil), C09DA (Angiotensin II receptor blockers (ARBs) and diuretics), C09DB01 (Valsartan and amlodipine), C09DB02 (Olmesartan medoxomil and amlodipine), C09DB07 (Candesartan and amlodipine), C09DX01 (Valsartan, amlodipine and hydrochlorothiazide), C09DX03 (Olmesartan medoxomil, amlodipine and hydrochlorothiazide)<br>– Other agents acting on the renin-angiotensin system: C03DA04 (Eplerenon), C09XA02 (Aliskiren), C09XA52 (Aliskiren and hydrochlorothiazide) |
| 2 | Cardiovascular | Proportion of patients with atrial fibrillation or atrial flutter and with risk factors for a thromboembolic event who received oral anticoagulation during the past 12 months. | Atrial fibrillation or flutter and at least one $CHA_2DS_2$-VASc score[a] ≥2 for men and ≥3 for women | At least one ATC code in group B01A (Antithrombotic agents) during the past 12 months |
| 3 | Cardiovascular | Proportion of CHD patients without other antithrombotic agents who received antiplatelet agents during the past 12 months. | CHD and no ATC code in groups B01AA (Vitamin K antagonists), B01AB (Heparin group), B01AE (Direct thrombin inhibitors), B01AF (Direct factor Xa inhibitors) | At least one ATC code in group B01AC (Platelet aggregation inhibitors excl. heparin) during the past 12 months |
| 4 | Cardiovascular | Proportion of CHD without diabetes and without CKD in stage G5 who received statins during the past 12 months. | CHD and none of the following:<br>– Diabetes | At least one ATC code in groups C10AA (HMG CoA reductase inhibitors), C01BA (Combinations of various lipid modifying agents), C10BX (Lipid |

| No. | Domain | Quality indicator description | Criteria for quality indicator eligibility (denominator population) | Criteria for quality indicator achievement (numerator population) |
|---|---|---|---|---|
| | | | – CKD and all eGFR values ever recorded ≥15 ml/min/1.73m$^2$ [1] | modifying agents in combination with other drugs) during the past 12 months |
| 5 | Cardiovascular | Proportion of CHD patients whose smoking status was documented during the past 12 months. | CHD | Non-empty smoking status field of the electronic record during the past 12 months |
| 6 | Endocrine | Proportion of diabetes patients who received at least 2 HbA1c measurements at least 4 months apart during the past 12 months. | Diabetes mellitus | At least two HbA1c measurements at least 120 days apart during the past 12 months |
| 7 | Endocrine | Proportion of diabetes patients who received an influenza vaccination during the last vaccination period (October to December). | Diabetes mellitus | Documentation of influenza vaccination in the vaccination field of the electronic record during the latest vaccination period ranging from October to December |
| 8 | Endocrine | Proportion of diabetes patients whose latest HbA1c during the past 12 months lied below an adapted treatment target. | Diabetes mellitus | At least one of the following during the past 12 months:<br>– Latest HbA1c ≤7%<br>– Age ≥65 years and at least one chronic conditions among hypertension, CHD, CKD, asthma, COPD, or heart failure (any of ICD-10 code I50 (Heart failure), ICPC-2 code K77 (Heart failure)) and latest HbA1c ≤8%[b] |
| 9 | Pulmonary | Proportion of asthma patients with active controller therapy (long-acting beta2 agonist and/or long-acting muscarinic antagonist) who received inhaled corticosteroids during the past 12 months. | Asthma and at least one ATC code in groups R03AC12 (Salmeterol), R03AC13 (Formoterol), R03AC18 (Indacaterol), R03AC19 (Olodaterol), R03AK06 (Salmeterol and fluticasone), R03AK07 (Formoterol and budesonide), R03AK08 (Formoterol and beclometasone), R03AK10 (Vilanterol and fluticasone fuorate), R03AK11 (Formoterol and fluticasone), R03AK14 (Indacaterol and mometasone), R03AL03 (Vilanterol and umeclidinium bromide), R03AL04 (Indacaterol and glycopyrronium bromide), R03AL06 (Olodaterol and tiotropium bromide), R03AL08 (Vilanterol, umeclidinium bromide and fluticasone furoate), R03AL09 (Formoterol, glycopyrronium bromide and | At least one ATC code in groups R03AK06 (Salmeterol and fluticasone), R03AK07 (Formoterol and budesonide), R03AK08 (Formoterol and beclometasone), R03AK10 (Vilanterol and fluticasone fuorate), R03AK11 (Formoterol and fluticasone), R03AK14 (Indacaterol and mometasone), R03AL08 (Vilanterol, umeclidinium bromide and fluticasone fuorate), R03AL09 (Formoterol, glycopyrronium bromide and beclometasone), R03AL11 (Formoterol, glycopyrronium bromide and budesonide), R03AL12 (Indacaterol, glycopyrronium bromide and mometasone), R03BA (Glucocorticoids) during the past 12 months |

| No. | Domain | Quality indicator description | Criteria for quality indicator eligibility (denominator population) | Criteria for quality indicator achievement (numerator population) |
|---|---|---|---|---|
| | | | beclometasone), R03AL11 (Formoterol, glycopyrronium bromide and budesonide), R03AL12 (Indacaterol, glycopyrronium bromide and mometasone), R03BB04 (Tiotropium bromide), R03BB05 (Aclidinium bromide), R03BB06 (Glycopyrronium bromide), R03BB07 (Umeclidinium bromide) | |
| 10 | Pulmonary | Proportion of COPD patients who received an influenza vaccination during the last vaccination period (October to December). | COPD | Documentation of influenza vaccination in the vaccination field of the electronic record during the latest vaccination period ranging from October to December |
| 11 | Pulmonary | Proportion of asthma and/or COPD patients whose smoking status was documented during the past 12 months. | Asthma and/or COPD | Non-empty smoking status field of the electronic record during the past 12 months |
| 12 | Renal | Proportion of CKD patients not in stage G5 whose latest BP during the past 12 months was <140/90 mmHg. | CKD and all eGFR values ever recorded ≥15 ml/min/1.73m$^2$ | Latest BP <140 mmHg systolic and <90 mmHg diastolic during the past 12 months |
| 13 | Renal | Proportion of CKD patients not in stage G5 who received at least one measurement of serum creatinine and BP during the past 14 months. | CKD and all eGFR values ever recorded ≥15 ml/min/1.73m$^2$ | At least one serum creatinine and at least one BP measurement in the past 14 months |
| 14 | Renal | Proportion of CKD patients not in stage G5 who received an angiotensin-converting enzyme inhibitor or an angiotensin receptor II blocker during the past 14 months. | CKD and all eGFR values ever recorded ≥15 ml/min/1.73m$^2$ | At least one ATC code in groups C09A (ACE inhibitors, plain), C09C (Angiotensin receptor II blockers (ARBs), plain) |

[a]The $CHA_2DS_2$-VASc Score was calculated from the following components [5]:

- Congestive heart failure (1 point): Any of ICD-10 code I50 (Heart failure), ICPC-2 code K77 (Heart failure).
- Hypertension (1 point): same definition as in the table on page 1 above.
- Age ≥75 year (2 points).
- Diabetes mellitus (1 point): same definition as in the table on page 1 above.
- Stroke/transient ischemic attack/thromboembolism (2 points): Any of ICD-10 codes G45 (Transient cerebral ischemic attacks and related syndromes), I63 (Cerebral infarction), I64 (Stroke, not specified as hemorrhage or infarction), I74 (Arterial embolism and thrombosis), ICPC-2 codes K89 (Transient cerebral ischemia), K90 (Stroke/cerebrovascular accident).
- Vascular disease (1 point): Any of ICD-10 codes I21 (Acute myocardial infarction), I22 (Subsequent myocardial infarction), I25.2 (Old myocardial infarction), I70.0 (Atherosclerosis of aorta), I70.2 (Atherosclerosis of arteries of extremities), ICPC-2 codes K75 (Acute myocardial infarction), K92 (Atherosclerosis/PVD).

- Age 65 to 74 years (1 point).
- Sex category (1 point for female sex).

[b]These targets were defined following the recommendations of the Swiss Society for Endocrinology and Diabetes (adapted optimal control), with adaptation to data availability in the FIRE database [6].

Abbreviations: ATC, Anatomic Therapeutic Chemical classification system; CHD, coronary heart disease; CKD, chronic kidney disease; COPD, chronic obstructive pulmonary disease; eGFR, estimated glomerular filtration rate; ICD-10, International Classification of Disease; ICPC-2, International Classification of Primary Care.

## References


1. Eknoyan G, Lameire N, Eckardt K, Kasiske B, Wheeler D, Levin A, et al. KDIGO 2012 clinical practice guideline for the evaluation and management of chronic kidney disease. Kidney Int Suppl. 2013;3(1):5-14.

2. Cosentino F, Grant PJ, Aboyans V, Bailey CJ, Ceriello A, Delgado V, et al. 2019 ESC Guidelines on diabetes, pre-diabetes, and cardiovascular diseases developed in collaboration with the EASD. Eur Heart J. 2020;41(2):255-323. doi: 10.1093/eurheartj/ehz486. PubMed PMID: 31497854.

3. Mancia G, Fagard R, Narkiewicz K, Redon J, Zanchetti A, Böhm M, et al. 2013 ESH/ESC guidelines for the management of arterial hypertension: the Task Force for the Management of Arterial Hypertension of the European Society of Hypertension (ESH) and of the European Society of Cardiology (ESC). Eur Heart J. 2013;34(28):2159-219. Epub 2013/06/19. doi: 10.1093/eurheartj/eht151. PubMed PMID: 23771844.

4. Federal Office of Public Health (FOPH). Health insurance: risk compensation Bern, Switzerland2024 [Cited 2024 June 20]. Available from: https://www.bag.admin.ch/bag/en/home/versicherungen/krankenversicherung/krankenversicherung-versicherer-aufsicht/risikoausgleich.html.

5. Hindricks G, Potpara T, Dagres N, Arbelo E, Bax JJ, Blomstrom-Lundqvist C, et al. 2020 ESC Guidelines for the diagnosis and management of atrial fibrillation developed in collaboration with the European Association for Cardio-Thoracic Surgery (EACTS): The Task Force for the diagnosis and management of atrial fibrillation of the European Society of Cardiology (ESC) Developed with the special contribution of the European Heart Rhythm Association (EHRA) of the ESC. Eur Heart J. 2021;42(5):373-498. doi: 10.1093/eurheartj/ehaa612. PubMed PMID: 32860505.

6. Gastaldi G, Lucchini B, Thalmann S, Alder S, Laimer M, Brandle M, et al. Swiss recommendations of the Society for Endocrinology and Diabetes (SGED/SSED) for the treatment of type 2 diabetes mellitus (2023). Swiss Med Wkly. 2023;153(4):40060. Epub 20230401. doi: 10.57187/smw.2023.40060. PubMed PMID: 37011604.
