# Supplementary material for: The effectiveness of a multi-domain electronic feedback report on the performance of quality indicators for chronic conditions: Protocol for a randomized controlled trial in general practice
Source: PLoS One. 2024 Nov 21;19(11):e0314360. doi: 10.1371/journal.pone.0314360 (PMC11581287; doi:10.1371/journal.pone.0314360)
Supplement: S3 Appendix — (PDF) [file pone.0314360.s003.pdf]

FIRE PROJECT

Feedbackreport

Reportingperiode: Juli 2023 – Juni 2024

Universität Zürich UZH

Kennzahlen

	Ihr Kollektiv	Differenz[1]	FIRE-Kollektiv[2]
Behandelte Patient*innen (n)	897	-5.4%	658
Konsultationen (n)	2’929	-2.3%	2’978

[1] Differenz zum Feedbackreport der Vorperiode; [2] Mediane Anzahl für das FIRE-Kollektiv in der Reportingperiode

Altersstruktur

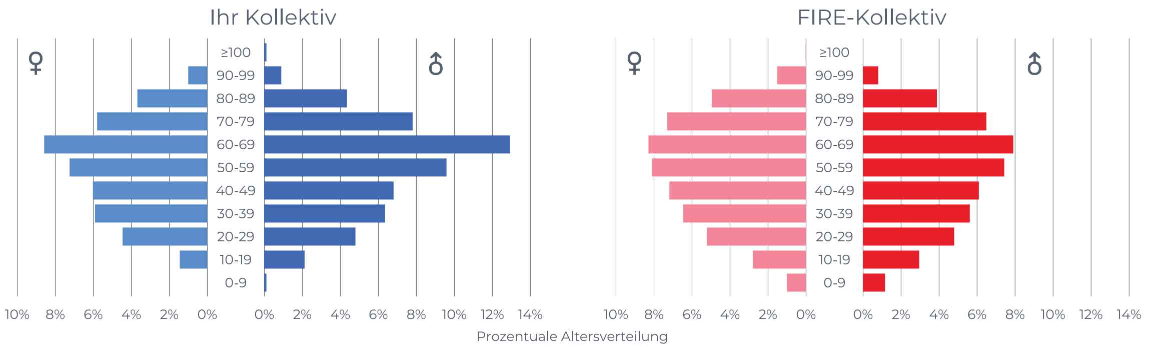

Geschlechterverteilung

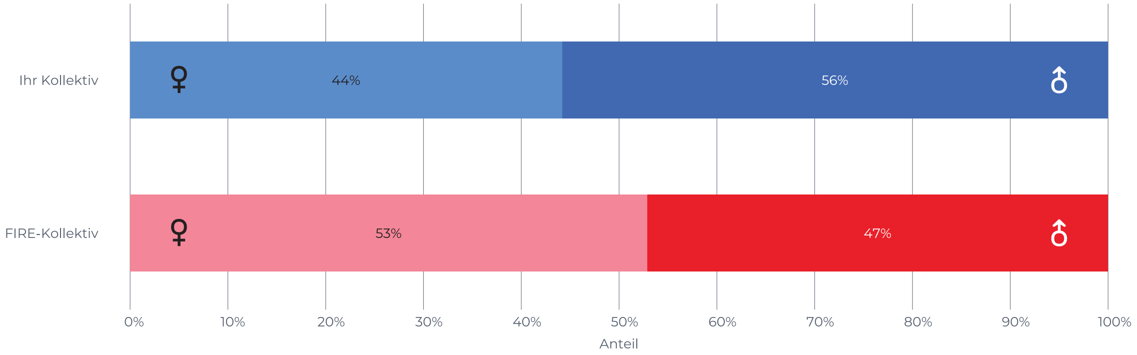

Klinisch-chemische Parameter

Parameter		Ihr Kollektiv	Diff.[2]	FIRE-Kollektiv
Gesamt-cholesterin *[mmol/l]*	n/n[1]	323	-12.0%	191
	Median	4.9	±0.0	5.0
	IQR	4.1–5.6	n/a	4.1–5.8
High Density Lipoprotein (HDL) *[mmol/l]*	n/n[1]	323	-12.0%	190
	Median	1.3	±0.0	1.5
	IQR	1.1–1.5	n/a	1.2–1.8
Low Density Lipoprotein (LDL) *[mmol/l]*	n/n[1]	323	-12.0%	190
	Median	2.9	+0.1	2.7
	IQR	2.3–3.5	n/a	2.0–3.5
Trigylceride *[mmol/l]*	n/n[1]	322	-12.0%	190
	Median	1.1	±0.0	1.3
	IQR	0.8–1.5	n/a	0.9–1.9
HbA1c *[%]*	n/n[1]	317	-6.5%	198
	Median	5.7	+0.1	5.6
	IQR	5.4–6.3	n/a	5.3–6.0
Glomeruläre Filtrationsrate (GFR) *[90ml/min/1.73m²]*	n/n[1]	452	-9.1%	312
	Median	88	-2	90
	IQR	73–101	n/a	74–103

[1] Mediane Anzahl für das FIRE-Kollektiv in der Reportingperiode; [2] Differenz zum Feedbackreport der Vorperiode
IQR: Interquartile range = Mittlere 50% Ihrer Kolleg*innen; n/a = nicht anwendbar

FIRE PROJECT

Feedbackreport

Reportingperiode: Juli 2023 – Juni 2024

Universität Zürich UZH

Blutdruck²

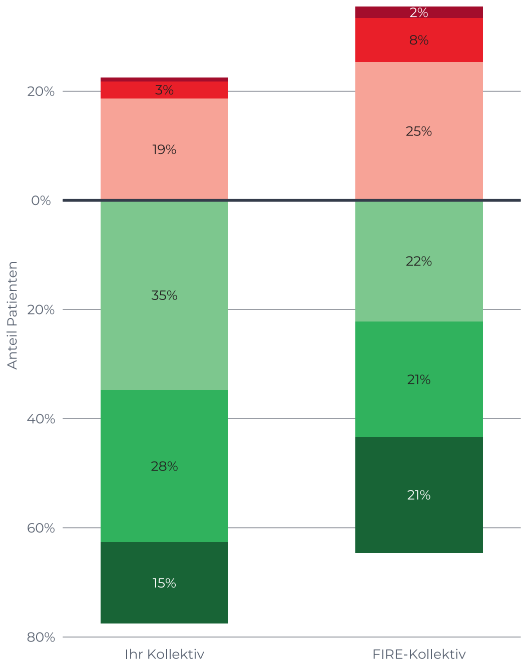

² Zuletzt gemessener Wert; Ihr Kollektiv: n = 610; FIRE-Kollektiv (Median): n = 292

- Optimaler Blutdruck <120 mmHg
- Normaler Blutdruck 120–129 mmHg
- Hochnormaler Blutdruck 130–139 mmHg
- Milde Hypertonie 140–159 mmHg
- Mittlere Hypertonie 160–179
- Schwere Hypertonie ≥180 mmHg

Chronische Krankheiten und Raucherstatus

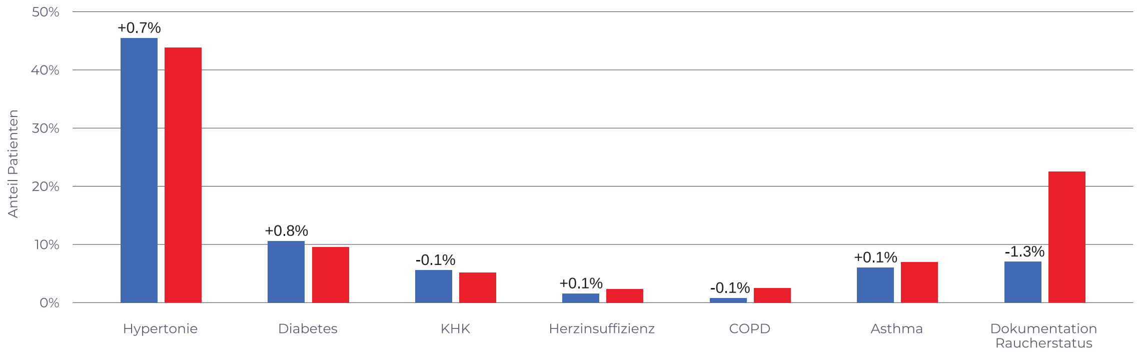

KHK: Koronare Herzkrankheiten
Prozentzahl über Ihrem Balken: Differenz zum Feedbackreport der Vorperiode

- Ihr Kollektiv n = 452
- FIRE-Kollektiv (Median) n = 292

Medikamente und Grippeimpfung

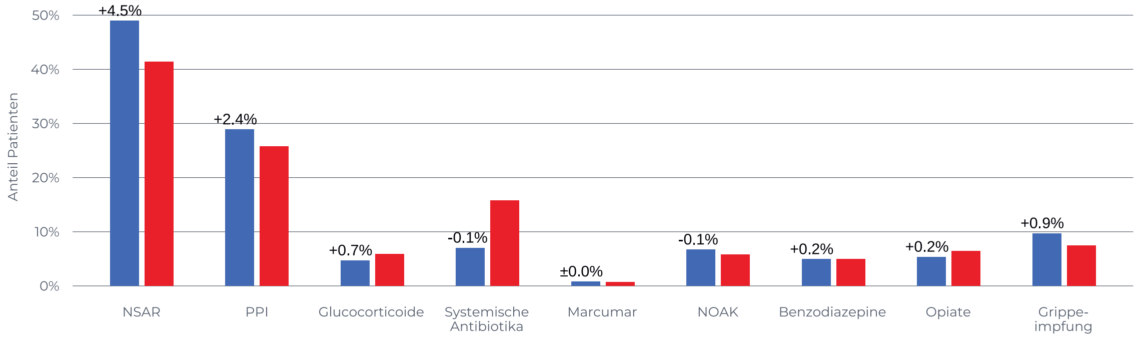

NSAR: Nicht steroidale Antirheumatika
PPI: Protonenpumpenhemmer
NOAK: Neue orale Antikoagulanzien
Prozentzahl über Ihrem Balken: Differenz zum Feedbackreport der Vorperiode

- Ihr Kollektiv n = 592
- FIRE-Kollektiv (Median) n = 393

Bodymass Index (BMI)

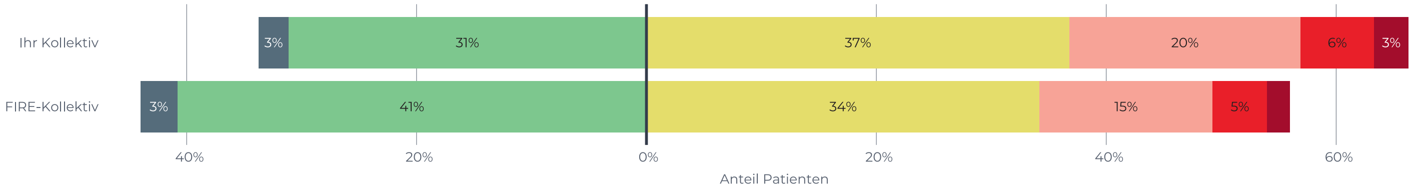

Ihr Kollektiv: n = 777; FIRE-Kollektiv (Median): n = 414

- BMI <18.5
- BMI 18.5–24.9
- BMI 25–29.9
- BMI 30–34.9
- BMI 35–39.9
- BMI ≥40

Anmerkung zum Raucherstatus und zur Grippeimpfung

FIRE erfasst die Daten wie folgt:

PIS	Raucherstatus	Grippeimpfung
Aeskulap	Problem-/Diagnoseliste, Risiken	Impfungen, Medikamente
curaMED	Problem-/Diagnoseliste, Risikofaktoren	Impfungen, Medikamente
pex II	Problem-/Diagnoseliste, Risikofaktoren	Impfungen, Neue Therapie/Medikamente
siMed	Problem-/Diagnoseliste, Noxen	Impfstatus, Medikation
tomedo	Problem-/Diagnoseliste	Medikamente
vitomed	Problem-/Diagnoseliste, Habituation, Anamnese-Art	Impfungen, Medikation
WinMed	Problem-/Diagnoseliste, Risikofaktoren	Impfungen, Medikamente
