# Supplementary material for: The effectiveness of a multi-domain electronic feedback report on the performance of quality indicators for chronic conditions: Protocol for a randomized controlled trial in general practice
Source: PLoS One. 2024 Nov 21;19(11):e0314360. doi: 10.1371/journal.pone.0314360 (PMC11581287; doi:10.1371/journal.pone.0314360)
Supplement: S4 Appendix — (PDF) [file pone.0314360.s004.pdf]

Feedback-Report

FIRE PROJECT

Universität Zürich[UZH]

Reportingperiode: Mai 2023–April 2024

Patientencharakteristika

Ihr Kollektiv

n=2051

Geschlecht

45%

55%

Prozentuale Geschlechterverteilung

Alter

Prozentuale Altersverteilung

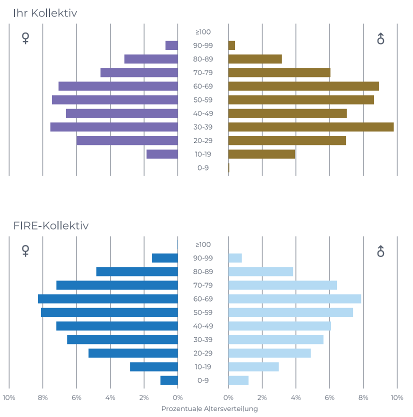

Body-Mass-Index (BMI)

Prozentuale BMI-Verteilung

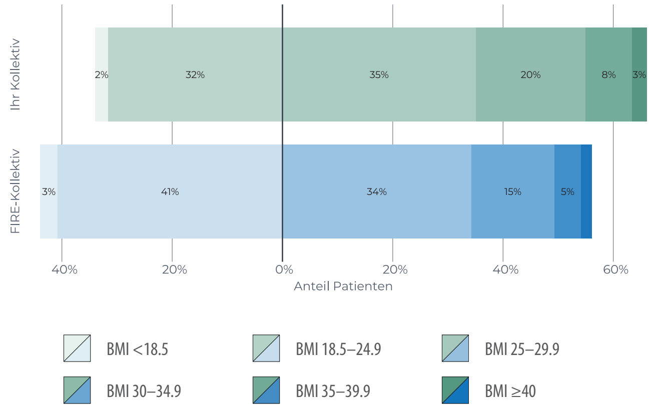

Kardiovaskuläre Erkrankungen

Hypertonie (n=571)

Kontrollierte oder resistente Hypertonie K1

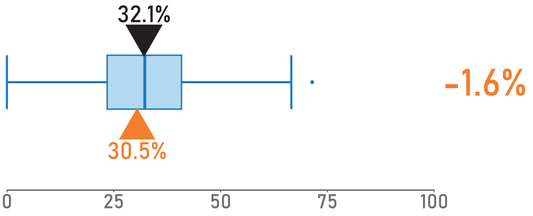

% Patienten mit Zielwert <140/90 mmHg oder mind. 3 Antihypertensiva

Vorhofflimmer/-flattern (n=69)

Risikofaktoren für Thromboembolisches Ereignis (TEE) K2

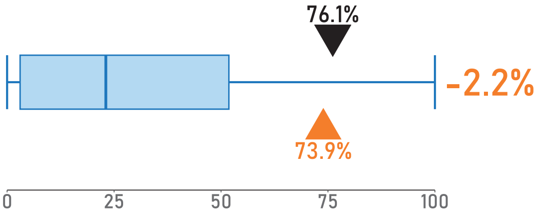

% Patienten mit Risikofaktoren für TEE in den letzten 12 Monaten unter OAK

Koronare Herzkrankheit

Thrombozytenaggregationshemmer K3

(n=93)

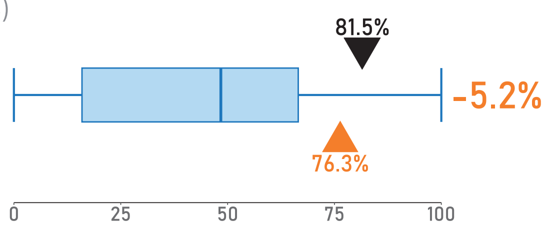

% Patienten mit Thrombozytenaggregationshemmern

Statine K4

(n=120)

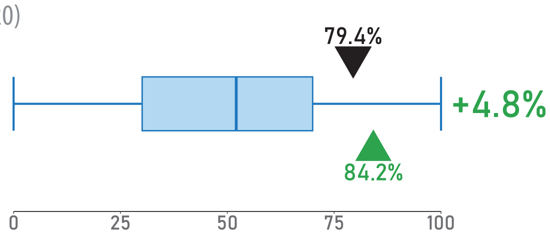

% Patienten mit Statinen

Dokumentierter Raucherstatus K5

(n=120)

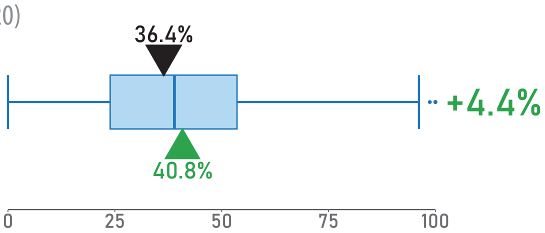

% Patienten mit dokumentiertem Raucherstatus

Endokrine Erkrankungen

Diabetes mellitus (n=137)

HbA1c-Messung
E1

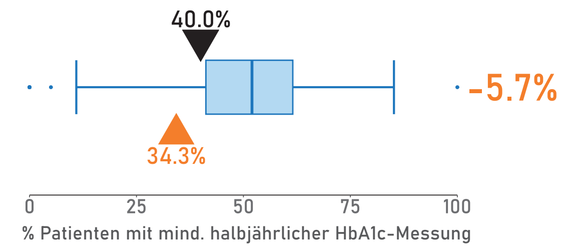

Grippeimpfung
E2

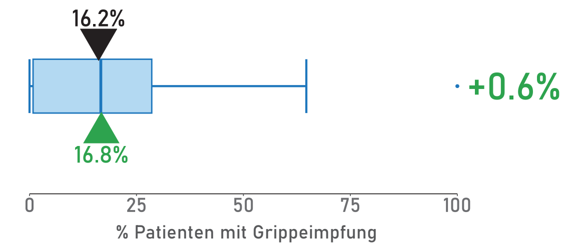

HbA1c-Zielwert gemäss SGED-Leitlinie
E3

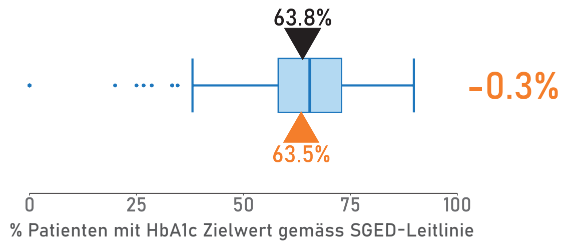

Lungenerkrankungen

Asthma (n=60)

Inhalatives Kortikosteroid & Controller
L1

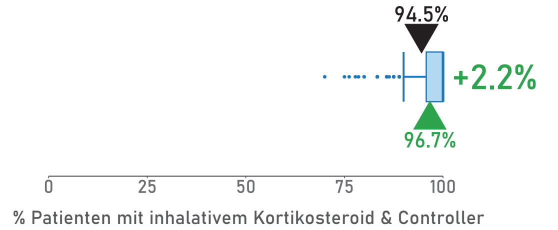

COPD (n=29)

Grippeimpfung
L2

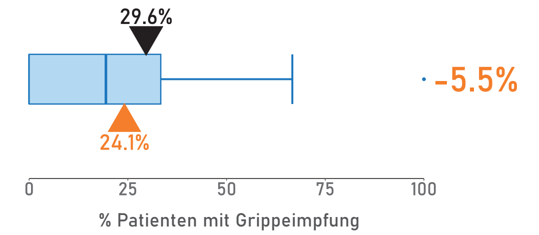

Asthma/COPD (n=120)

Dokumentierter Raucherstatus
L3

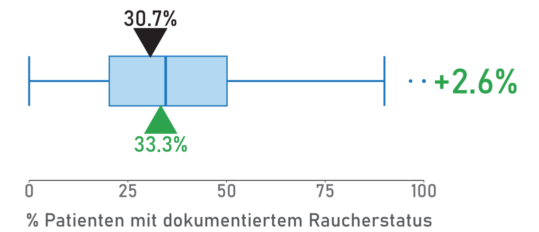

Nierenerkrankungen

Chronische Nierenerkrankung

Serumkreatinin- & Blutdruckmessung
(n=87)
N1

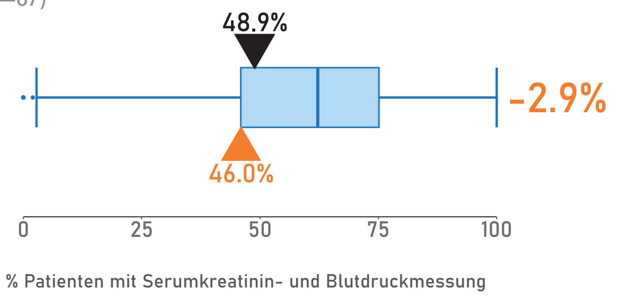

Blutdruck <140/90 mmHg
(n=87)
N2

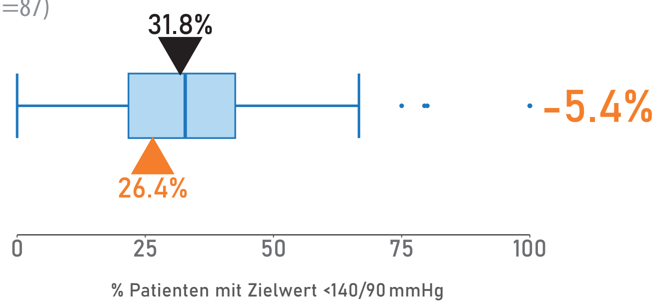

Verschreibung RAAS-Blocker
(n=91)
N3

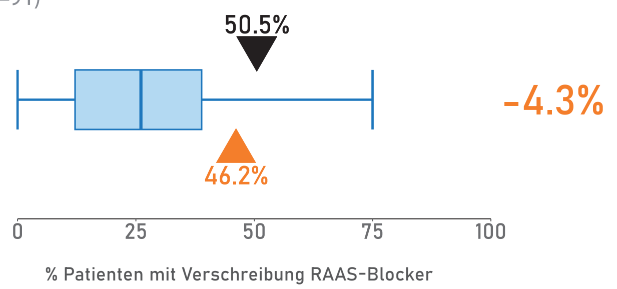

FIRE PROJECT

Feedback-Report: Zusatzblatt

Universität Zürich UZH

Boxplot einfach erklärt

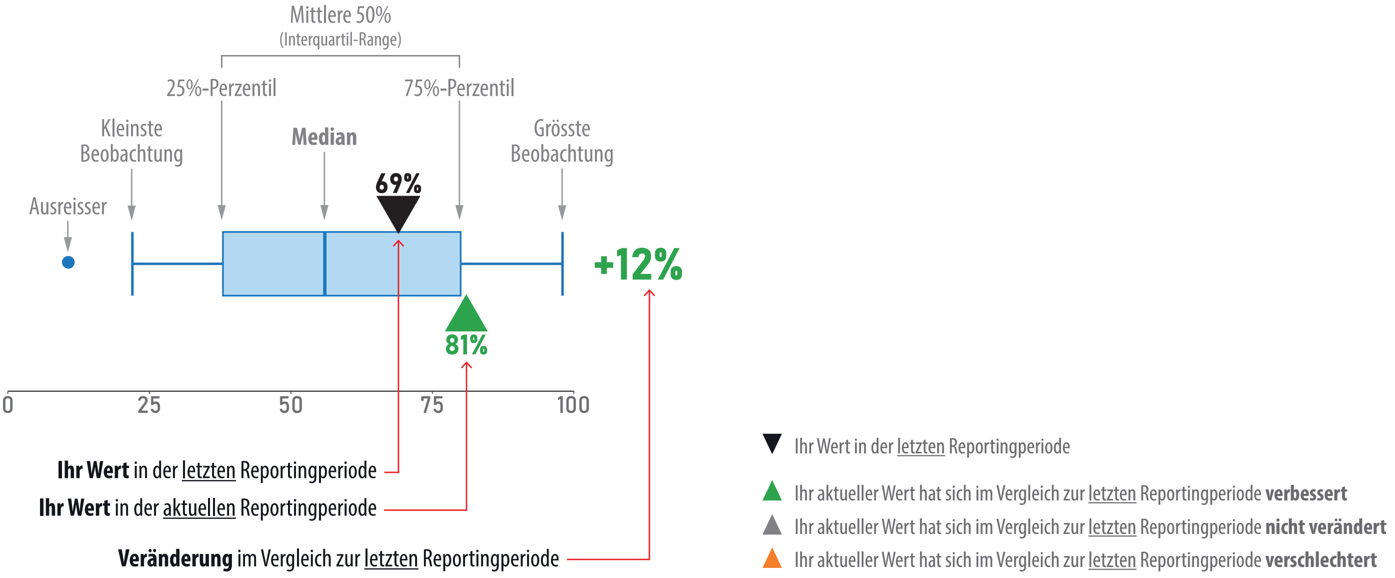

Erarbeitung der Qualitätsindikatoren

Das FIRE-Research-Team recherchierte (inter)national verfügbare Indikatoren für die dargestellten Erkrankungen der jeweiligen Krankheitsdomänen (kardiovaskuläre Erkrankungen, endokrine Erkrankungen, Lungenerkrankungen, Nierenerkrankungen und Infektiologie) und überprüfte diese auf ihre Abbildbarkeit in FIRE. Auf dieser Grundlage wurden 57 Indikatoren ausgewählt, die ein zweistufiges Bewertungsverfahren durchlaufen haben. Zuerst wurden mit einem Experten der entsprechenden Krankheitsdomäne die jeweiligen Indikatoren besprochen, angepasst, gelöscht oder neue Indikatoren hinzugefügt. In einem zweiten Schritt wurden alle verbleibenden 33 Indikatoren mit sieben Hausärzt*innen bewertet und die finalen 15 Indikatoren festgelegt.

Berechnung der Qualitätsindikatoren

Nr.	Erkrankungen	Qualitätsindikator	Zähler	Nenner	Referenzen
K1	Hypertonie	Anteil der Patienten mit Hypertonie mit einem Blutdruck < 140/90 mmHg (primäres Behandlungsziel) oder mindestens drei verordneten Antihypertensiva verschiedener Arzneimittelklassen	Anzahl Hypertonie-Patienten[1,a], bei welchen der letzte Blutdruck unter 140/90 mmHg lag oder die bei einem höheren Wert zuletzt zeitgleich mindestens drei Antihypertensiva verschiedener Wirkstoffklassen (in Form von Mono- oder entsprechenden Kombinationspräparaten) erhielten	Anzahl aller Hypertonie-Patienten[1,a]	Adaptiert gemäss QiSA Band C3 «Bluthochdruck» (V2.0, Indikator 7)
K2	Vorhofflimmern/ -flattern	Anteil der Patienten mit Vorhofflimmern oder Vorhofflattern und mit Risikofaktoren für ein thromboembolisches Ereignis innerhalb der letzten 12 Monate, die eine orale Antikoagulation erhalten	Anzahl Patienten mit Vorhofflimmern/-flattern[2] und mit CHA2DS2-VASc Score ≥2 für Männer und ≥3 für Frauen, die eine orale Antikoagulation[b] erhalten	Anzahl aller Patienten mit Vorhofflimmern/-flattern[2] und CHA2DS2-VASc Score ≥2 für Männer und ≥3 für Frauen mit einer oralen Antikoagulation[b]	Gemäss NHS QOF (Seite 26). Mit FIRE-Daten abbildbare Risikofaktoren umfassen: Alter, Geschlecht, chron. Herzinsuffizienz, Hypertonie, Diabetes mellitus Später erfassbare Risikofaktoren: Arteriosklerose, St.n. Stroke, TIA
K3	KHK	Anteil der KHK-Patienten, die Thrombozytenaggregationshemmer erhalten	Anzahl KHK-Patienten[3] mit Thrombozytenaggregations-hemmern	Anzahl aller KHK-Patienten[3]	Adaptiert gemäss QiSA Band C7 «Koronare Herzkrankheit» (V2.0, Indikator 5)
K4	KHK	Anteil der KHK-Patienten, die Statine erhalten	Anzahl KHK-Patienten[3,c] mit Statin(en)	Anzahl aller KHK-Patienten[3,c]	Adaptiert gemäss QiSA Band C7 «Koronare Herzkrankheit» (V2.0, Indikator 8)
K5	KHK	Anteil der KHK-Patienten mit dokumentiertem Raucherstatus	Anzahl KHK-Patienten[3] mit dokumentiertem Raucherstatus[4]	Anzahl aller KHK-Patienten[3]	Adaptiert gemäss QiSA Band C7 «Koronare Herzkrankheit» (V2.0, Indikator 13)

Nr.	Erkrankungen	Qualitätsindikator	Zähler	Nenner	Referenzen
E1	Diabetes mellitus	Anteil der Diabetiker mit mindestens halbjährlicher HbA1c-Bestimmung	Anzahl Patienten mit vorbestehendem Diabetes mellitus[5] (vor Reportingperiode) mit mindestens halbjährlicher HbA1c-Bestimmung	Anzahl aller Patienten mit vorbestehendem Diabetes mellitus[5] (vor Reportingperiode)	Gemäss SGED-Leitlinien 2023 und QiSA Band C2 «Diabetes Mellitus 2» (V2.0, Indikator 2)
E2	Diabetes mellitus	Anteil der Diabetiker mit Grippeimpfung	Anzahl Patienten mit vorbestehendem Diabetes (vor Reportingperiode) mellitus[5] mit Grippeimpfung	Anzahl aller Patienten mit vorbestehendem Diabetes mellitus[5] (vor Reportingperiode)	Gemäss der Empfehlung der Abteilung Übertragbare Krankheiten des BAG wird Personen ab dem Alter von 6 Monaten mit Diabetes mellitus die Grippeimpfung empfohlen.
E3	Diabetes mellitus	Anteil der Diabetiker mit einem HbA1c-Wert, der unter dem SGED-Leitlinien-Zielwert liegt (optimale Einstellung, angepasst)[5]	Anzahl Patienten mit vorbestehendem Diabetes mellitus[5] (vor Reportingperiode) mit letztem HbA1c-Wert unter dem SGED-Leitlinien-Zielwert	Anzahl aller Patienten mit vorbestehendem Diabetes mellitus[5] (vor Reportingperiode)	Der HbA1c-Zielwert sollte bei den meisten Patienten ≤7% betragen. Für Patienten ≥65 Jahre mit Multimorbidität ist ein höherer HbA1c-Zielwert von ≤8% sinnvoll. Diese Zielwerte werden in den SGED-Leitlinien (optimale Kontrolle) entsprechend den Möglichkeiten der FIRE-Datenbank angepasst.
L1	Asthma	Anteil der ärztlich diagnostizierten und mit Controller (LAMA oder LABA) behandelten Asthma-Patienten, die gleichzeitig mit inhalaltivem Kortikosteroid (ICS) behandelt werden	Anzahl Asthma-Patienten[3] mit ICS & Controller	Anzahl aller Asthma-Patienten[3] mit ICS & Controller oder nur Controller	Adaptiert gemäss QiSA Band C1 «Asthma/COPD» (V2.0, Indikator 2)
L2	COPD	Anteil der COPD-Patienten mit Grippeimpfung	Anzahl Patienten mit vorbestehender COPD[3] (vor Reportingperiode) mit Grippeimpfung	Anzahl aller Patienten mit vorbestehender COPD[3] (vor Reportingperiode)	Adaptiert gemäss QiSA Band C1 «Asthma/COPD» (V2.0, Indikator 9)
L3	Asthma/COPD	Anteil der Asthma- und COPD-Patienten mit dokumentiertem Raucherstatus	Anzahl Asthma/COPD-Patienten[3] mit dokumentiertem Raucherstatus[4]	Anzahl aller Asthma/COPD-Patienten[3]	Gemäss Experten- und Hausärzte-Konsensus
N1	Chronische Niereninsuffizienz	Anteil der chron. Niereninsuffizienz-Patienten mit Messung von Serumkreatinin und Blutdruck in den letzten 14 Monaten	Anzahl CKD-Patienten[6] (mind. 2 Monate vor Reportingperiode identifiziert) mit Serumkreatinin- und Blutdruckmessung in den 14 Monaten	Anzahl CKD-Patienten[6] (mind. 2 Monate vor Reportingperiode identifiziert) mit Stadium G2–G4	Adaptiert gemäss: van Gelder et al. (Scand J Prim Health Care. 2016; 34(1):73–80) und Tu et al. (CMAJ Open. 2017; 5(1):E74–e81)
N2	Chronische Niereninsuffizienz	Anteil der chron. Niereninsuffizienz-Patienten mit einem letzten Blutdruck <140/90 mmHg	Anzahl CKD-Patienten[6] (mind. 2 Monate vor Reportingperiode identifiziert) mit letzter Blutdruckmessung <140/90 mmHg	Anzahl CKD-Patienten[6] (mind. 2 Monate vor Reportingperiode identifiziert) mit Stadium G2–G4	Adaptiert gemäss: Tu et al. (CMAJ Open. 2017; 5(1):E74–e81)
N3	Chronische Niereninsuffizienz	Anteil der chron. Niereninsuffizienz-Patienten, die in den letzten 14 Monaten einen ACE-Hemmer oder Angiotensin II-Rezeptorblocker verschrieben bekommen haben	Anzahl CKD-Patienten[6] (mind. 2 Monate vor Reportingperiode identifiziert) mit Verschreibung RAAS-Blocker[7] in den 14 Monaten	Anzahl CKD-Patienten[6] (mind. 2 Monate vor Reportingperiode identifiziert) mit Stadium G2–G4 und Albuminurie >30mg/ml/24h	Adaptiert gemäss: Nash et al. (Can J Kidney Health Dis. 2017; 4:20543581177) und Bello et al. (JAMA Netw Open. 2019; 2(9):e1910704)

[1] Bestimmt mittels Medikamente, Vitalparameter, ICPC-2
[2] Gemäss NHS QOF Seite 26. Mit FIRE-Daten abbildbare Risikofaktoren umfassen: Alter, Geschlecht, chron. Herzinsuffizienz, Hypertonie, Diabetes mellitus
Später erfassbare Risikofaktoren: Arteriosklerose, St.n. Stroke oder TIA
Limitation: Patienten mit Kontraindikationen können nicht bestimmt werden
[3] Bestimmt mittels Freitextdiagnose/ICPC-2/ICD-10
[4] Bestimmt mittels Freitextdiagnose/ICPC-2/ICD-10 oder strukturiertes Eingabefeld in eKG
[5] Bestimmt mittels Medikamente, Laborparameter, ICPC-2
[6] Bestimmt mittels Laborparameter und Freitextdiagnose/ICPC-2/ICD-10
[7] ACE-Hemmer oder Angiotension II-Rezeptorblocker

Ausschlusskriterien:
[a] Patienten ≥80 Jahre
[b] anderes Antikoagulans (z.B. bei gleichzeitigem Vorhofflimmern)
[c] Patienten (≥73 Jahre) mit zusätzlich ischämischer Kardiomyopathie oder Patienten mit Typ 2-Diabetes und terminaler Niereninsuffizienz

Anmerkung zum Raucherstatus und zur Grippeimpfung

FIRE erfasst die Daten wie folgt:

PIS	Raucherstatus	Grippeimpfung
**Aeskulap**	Problem-/Diagnoseliste, Risiken	Impfungen, Medikamente
**curaMED**	Problem-/Diagnoseliste, Risikofaktoren	Impfungen, Medikamente
**pex II**	Problem-/Diagnoseliste, Risikofaktoren	Impfungen, Neue Therapie/Medikamente
**siMed**	Problem-/Diagnoseliste, Noxen	Impfstatus, Medikation
**tomedo**	Problem-/Diagnoseliste	Medikamente
**vitomed**	Problem-/Diagnoseliste, Habituation, Anamnese-Art	Impfungen, Medikation
**WinMed**	Problem-/Diagnoseliste, Risikofaktoren	Impfungen, Medikamente
