# Supplementary material for: The effectiveness of a multi-domain electronic feedback report on the performance of quality indicators for chronic conditions: Protocol for a randomized controlled trial in general practice
Source: PLoS One. 2024 Nov 21;19(11):e0314360. doi: 10.1371/journal.pone.0314360 (PMC11581287; doi:10.1371/journal.pone.0314360)
Supplement: S5 Appendix — (PDF) [file pone.0314360.s005.pdf]

Brief description of the project

Please write in English or in the official local language of the competent Ethics Committee to which you are submitting the clarification of responsibility.

Texts in *italic blue* are instructions and should be deleted.

Name of the project:	Randomized controlled trial of the effectiveness of feedback reporting on quality of patient care in Swiss general practice
Institution:	Institute of Primary Care, University and University Hospital Zurich, Pestalozzistrasse 24, 8091 Zurich, Switzerland
Name of the applicant:	Prof. Dr. med. Thomas Rosemann, Ph.D.
Submission date:	06.09.2023
Objective(s)/Aim:	The aim of this study is to determine whether the implementation of a feedback report (FBR) with or without quality indicators (QIs) leads to an increase in the quality of patient care in Swiss primary care.
Outcome/Endpoints	As the **primary outcome**, we will use an overall QI performance score (OPS) calculated by dividing the number of instances of all QIs in which recommended care was delivered by the number of instances of all QIs in which patients were eligible for such care at 12 months.<br><br>**Secondary outcomes** will be a specific domain QI performances score (DPS) for each individual domain (cardiovascular, endocrinological, infectiological, pulmonary, or renal), calculated by dividing the number of instances of the specific domain QI in which recommended care was delivered by the number of instances of the specific domain QI in which patients were eligible for such care at 12 months.<br><br>An **additional secondary outcome** will be an individual performance score (IPS) per general practitioner (GP). This secondary outcome will be assessed at baseline and at 12 months.
Project design and procedures	**Study design**<br>We plan a two-arm randomized controlled trial (RCT) in Swiss primary care: one group will receive "comprehensive" feedback consisting of evidence based QIs, while the other group will receive the standard "simple" feedback.<br><br>**Intervention**<br>Both study arms will receive a personalized FBR delivered bi-monthly via email, each covering the previous 12 months, and the FBRs will differ between study arms. The intervention arm will receive a comprehensive FBR consisting of 15 QIs (QI-FBR) that cover the following domains: cardiovascular, endocrinological,

	infectiological, pulmonary, or renal. The QI-FBR will provide detailed information on the performance of each participating GP, including performance against their peers (benchmarking) and changes from the previous report.<br><br>GPs assigned to the control arm will receive a standard "simple" FBR focused on demographic and clinical information (DC-FBR). This report will provide information solely on the number of patients treated, patient demographic data, blood pressure distributions as well as numbers of selected laboratory parameters, chronic diseases and medication. Additionally, unlike the intervention group, the GPs in the control group will not have access to peer comparisons.<br><br>**Data source**<br>The source of the study data will be the FIRE research database, in which GPs voluntarily provide anonymized, structured routine medical data from their electronic medical records (www.fireproject.ch). It includes consultations, administrative, clinical (vital signs), laboratory and medication data, ICPC-2 (International Classification of Primary Care, 2nd edition) codes as well as diagnosis lists in free-text format of patients. Data is collected automatically on a daily basis from the participating GPs using an application programming interface (API) developed specifically for the FIRE project. Free-text diagnoses relevant to this study are classified according to ICD-10 (International Statistical Classification of Diseases and Related Health Problems, 10th revision).
Does the project involve the participation of persons	YES___ NO ☒<br><br>**Describe the population studied:**<br>The participants will be GPs who have formally enrolled and actively participate in the FIRE project. In brief, to be eligible, GPs must have been working in general practice for at least one year prior to the study and must be board certified. To assess patient-level outcomes, patients from the FIRE database will be included if they are over 18 years of age. As described above, patient data is routinely collected by the GPs and automatically uploaded to the FIRE database.
Is health-related personal data and / or biological material used and analysed in the project?	YES ☒ NO___<br><br>**If YES: number of data sets and number of samples:**<br>The source of the study data will be the FIRE database and data is collected automatically on a daily basis from the participating GPs (see also "Project design and procedures"). After each patient consultation, anonymized data will be exported on a per-patient basis. The GPs do not have to do anything actively.
Is the health-related personal data and / or biological material **coded**?	YES___ NO ☒

Is the health-related personal data and / or biological material **anonymized**?	YES ☒ NO___<br><br>**If YES, describe the process of anonymization of the health-related personal data and /or biological sample:**<br>The patient ID in the electronic medical record is automatically provided with a hash value ("hashed") by the export software of the software manufacturer using a special algorithm. This means that the original patient ID is specially changed so that it can no longer be "decrypted" or restored. Under these circumstances, re-identification of a patient is no longer possible.<br><br>**If YES, indicate who conducted the anonymization of the health-related personal data and /or biological sample. If this is not known explain:**<br>Anonymization is performed by the algorithm (see above) in the physician's respective electronic medical record.
Will this project generate generalizable knowledge?	YES___ NO ☒
Does the project meet the criteria of a quality assurance and quality control project of the guidance document «Quality assurance, or research project subject to approval?»	YES ☒ NO___<br><br>**Criterion 1: Goal of the project**<br>Feedback reports are recognized interventions that lead to potentially important improvements in the quality of care in health care and are one of the most widely used and promising interventions for implementing research and guidelines in practice. Further, the standard "simple" feedback has already been established in FIRE and therefore represents the "standard" or "usual" care within the FIRE participants (see also "Project design and procedures").<br><br>**Criterion 2: Purpose of the project**<br>The purpose of this study is to determine whether the implementation of a feedback report with or without quality indicators leads to an increase in the quality of patient care<br><br>**Criterion 3: Generalizability of knowledge**<br>The results of this study will not generate generalizable knowledge but refer only to the quality of care in Swiss general practice.<br><br>**Criterion 4: Project methodology**<br>The participants will be GPs who have formally enrolled and actively participate in the FIRE project. Data (on all patients) is collected automatically on a daily basis from the participating GPs and is a part of the patient management.<br><br>**General:**<br>It should be possible to publish the results in a scientific journal for quality assurance purposes.

Any additional information about the project relevant for evaluation:	The FIRE project is supported by a grant from the Federal Quality Commission (FQC) of the Federal Office of Public Health (FOPH) which funds national quality development projects (Gewährte Finanzhilfen (admin.ch)).
Has/have similar project(s) been submitted to the EC?	YES___ NO ☒
