# Supplementary material for: The effectiveness of a multi-domain electronic feedback report on the performance of quality indicators for chronic conditions: Protocol for a randomized controlled trial in general practice
Source: PLoS One. 2024 Nov 21;19(11):e0314360. doi: 10.1371/journal.pone.0314360 (PMC11581287; doi:10.1371/journal.pone.0314360)
Supplement: S6 Appendix — (PDF) [file pone.0314360.s006.pdf]

Details of the Power Analysis

In this document, we describe the approach used for the power analysis mentioned in the main text of the protocol. We based our calculations on a dataset available on 31 December 2023 derived from 64 GPs who met the eligibility criteria for the study, and assessed their QI performance during the period from 1 January 2023 to 31 December 2023. We used a model-based simulation to determine the statistical power at the 0.05 significance level (α) under different assumptions for the effect of the QI-FBR and for different GP sample sizes. Table 1 summarizes the characteristics of the sample.

**Characteristic**	**Value**<br>***n* = 64**
Overall denominator population size, median (IQR)	930 (598 to 1213)
PS_O, median (IQR)	35.6% (28.6% to 40.7%)
Female, *n* (%)	43 (51.2)
Workload in consultations per working week, median (IQR)	48.0 (35.8 to 71.0)

Table 1: Characteristics of the sample of GPs available for the power analysis. The overall denominator population size corresponds to the sum of the denominator population sizes of all QIs. Abbreviations: IQR, interquartile range; PS_O, overall performance score.

Model Assumptions

As described in the protocol, the overall performance score (PS_O) is defined as the weighted average of all quality indicator performance scores (PS_{QI}), where the weights are proportional to the denominator population sizes of the respective QIs. Equivalently, the PS_O of a GP can be obtained by dividing the *overall numerator population size*, defined as the sum of the numerator population sizes across all QIs, by the *overall denominator population size*, defined as the sum of the denominator population sizes across all QIs. This equivalence justifies treating overall QI performance by considering the overall numerator population size as a binomially distributed variable on level of the single GP, with the overall denominator population size as the total number of trials and a success probability determined by the GP's characteristics and type of FBR.

Following this argument, we will use a mixed-effects binomial regression model to analyze the primary study outcome PS_O. Each GP will contribute two observations, one for the baseline period and one for the follow-up period. The model will account for the two observations at the GP level and for clustering of GPs within practices through appropriate random effects. In addition to fixed effects for the general effect of FBRs (any of the QI-FBR or the P-FBR in the follow-up period versus no FBR in the baseline period) and for the study arm during the follow-up period (QI-FBR versus P-FBR), the model will include fixed-effect adjustment for the variables used for balanced randomization (GP sex and consultation workload). Accordingly, we based our simulation on a model with the following parameters:

- Fixed-effect intercept: β_0
- Fixed-effect coefficient for the general FBR effect (any of the QI-FBR or the P-FBR in the follow-up period versus no FBR in the baseline period): β_{FBR}
- Fixed-effect coefficient for the differential QI-FBR effect (QI-FBR versus P-FBR): β_{QI}
- Fixed-effect coefficient for sex (male versus female): β_{sex}
- Fixed-effect coefficients for consultation workload (2nd, 3rd, and 4th quartile versus 1st quartile): β_{Q2}, β_{Q3}, β_{Q4}
- Variance of GP-level random intercepts: σ^2_{GP}
- Variance of practice-level random intercepts: σ^2_{PR}

Let g be an index for the GP and t be a dummy variable for the evaluation period, where $t = 0$ corresponds to the baseline period and $t = 1$ to the follow-up period. We assumed the linear predictor to have the form

$$\eta_{gt} = \beta_0 + \beta_{\text{FBR}} \cdot t + \beta_{\text{QI}} \cdot \text{arm}(g) \cdot t + \beta_{\text{sex}} \cdot \text{sex}(g) + \sum_{j=2,3,4} \beta_{\text{Q}j} \cdot \text{workload}_j(g) + u_g + v_{\text{PR}(g)}, \qquad (1)$$

where $\text{arm}(g)$ is an indicator variable for the study arm (0 for the P-FBR arm and 1 for the QI-FBR arm), $\text{sex}(g)$ is a dummy variable for sex (0 for female and 1 for male), and $\text{workload}_j(g)$, $j = 2,3,4$ are indicator variables for the 2nd, 3rd, and 4th workload quartile, respectively. In addition, we assumed normally distributed random intercepts at the GP and practice level, i.e., independent terms $u_g \sim N(0, \sigma^2_{\text{GP}})$ and

$v_{\mathrm{PR}(g)} \sim N(0, \sigma^2_{\mathrm{PR}})$, where $\mathrm{PR}(g)$ is an index indicating the practice of the g-th GP. We used a logit link function to derive the corresponding probability of overall QI achievement,

$$p_{gt} = \mathrm{logit}^{-1}(\eta_{gt}). \quad (2)$$

For each GP, we assumed an overall denominator population size d_g that, for simplicity, did not vary between evaluation periods. The overall numerator population size n_{gt} of the g-th GP during the evaluation period t was then assumed to follow a binomial distribution with the total number of trials given by d_g and with the probability of success given by p_{gt}:

$$n_{gt} \sim \mathrm{Binom}(d_g, p_{gt}). \quad (3)$$

The exponentiated coefficients $\exp(\beta_{\mathrm{FBR}})$ and $\exp(\beta_{\mathrm{QI}})$ therefore indicate the odds ratios (ORs) for the general FBR effect (FBR versus no FBR) and the QI-FBR effect (QI-FBR versus P-FBR) on QI achievement in a single QI instance. The primary outcome, change in the $\mathrm{PS_O}$ between the baseline and follow-up period, is then reflected in the value of β_{QI} or $\exp(\beta_{\mathrm{QI}})$.

For our simulation, we assumed an OR of 1.09 for the QI-FBR effect ($\beta_{\mathrm{QI}} = \log(1.09)$), which corresponds to an absolute increase in the overall QI performance across all GPs in the available dataset of 2 percentage points (observed at 35.6%). For the general FBR effect, we examined the values $\beta_{\mathrm{FBR}} = \log(1.0), \log(1.3)$, and $\log(2.0)$, corresponding to the range of ORs observed in a previous study with electronic FBRs in the FIRE project [1]. For the remaining model parameters, we used estimates obtained by fitting a mixed-effects binomial regression model to the available dataset, using a structure analogous to (1), but without the terms relating to t and $\mathrm{arm}(g) \cdot t$. These estimates are summarized in Table 2.

Fixed-effect coefficient	Coefficient estimate (standard error)
β_0	-0.521 (0.092)
β_{sex}	0.047 (0.079)
β_{Q2}	-0.189 (0.103)
β_{Q3}	-0.097 (0.113)
β_{Q4}	-0.195 (0.136)
**Random intercept variance**	**Variance estimate (standard deviation estimate)**
σ^2_{GP}	0.053 (0.231)
σ^2_{PR}	0.078 (0.280)

Table 2: Estimates of the model parameters used for the power calculation, derived from a dataset of 64 GPs eligible for the study by 31 December 2023.

Simulation

We conducted our power analysis for sample sizes of $N = 50$, 75, and 100 GPs. With three sample sizes and three assumptions for β_{FBR}, we ran a total of 9 simulations with 5000 iterations each. For each value of β_{FBR} and of the sample size N, we performed the following steps in each iteration:

1) Generate a bootstrap sample of N GPs $g = 1, \ldots, N$ from the available dataset, including their practice $\mathrm{PR}(g)$, sex, workload, and size of denominator population d_g.
2) For each distinct practice $\mathrm{PR}(g)$ occurring among the GPs of the sample, generate a random intercept $v_{\mathrm{PR}(g)} \sim N(0, \sigma^2_{\mathrm{PR}})$.
3) For each GP g in the sample, generate a random intercept $u_g \sim N(0, \sigma^2_{\mathrm{GP}})$.
4) Randomize the GPs into the QI-FBR and the P-FBR arms using a 1:1 allocation ratio.
5) For each GP g in the sample, generate a numerator population size $n_{g,t=0}$ for the baseline period and a numerator population size for the follow-up period $n_{g,t=1}$:
    a. Calculate $p_{g,t=0}$ and $p_{g,t=1}$ according to (1) and (2).
    b. Generate $n_{g,t=0} \sim \mathrm{Binom}(d_g, p_{g,t=0})$ and $n_{g,t=1} \sim \mathrm{Binom}(d_g, p_{g,t=1})$ as in (3).
6) Fit a binomial regression model on the simulated dataset using the following specifications according to the structure of (1)-(3):
    a. Number of successes: n_{gt}.
    b. Number of failures: $d_g - n_{gt}$.
    c. Fixed-effect covariates: t, $\mathrm{arm}(g) \cdot t$, $\mathrm{workload}_j(g)$, $j = 2,3,4$, $\mathrm{sex}(g)$.
    d. Random effects: normally distributed random intercepts at GP and practice level.

7) Extract the coefficient estimate for the term $\mathrm{arm}(g) \cdot t$ as the estimate $\hat{\beta}_{\mathrm{QI}}$ of the QI-FBR effect from the fitted model, together with its standard error $\mathrm{SE}_{\mathrm{QI}}$.
8) Determine if $\hat{\beta}_{\mathrm{QI}}$ is significantly different from 0, i.e., whether $|\hat{\beta}_{\mathrm{QI}}/\mathrm{SE}_{\mathrm{QI}}| > z_{0.975}$, where $z_{0.975}$ is the 0.975-quantile of the standard normal distribution.

For each combination of β_{FBR} and N, the power was then calculated as the proportion of iterations that achieved statistical significance in step 7).

We conducted the simulation with the statistical software R (R Foundation for Statistical Computing, Vienna, Austria) and used the lme4 package for mixed-effects model fitting [2, 3]. The results of the simulation are presented in Table 3.

OR of FBR versus no FBR ($\exp(\beta_{\mathrm{FBR}})$)	Sample size	Power, %
1.0	50	88.1
1.0	75	96.7
1.0	100	99.4
1.3	50	88.1
1.3	75	97.4
1.3	100	99.6
2.0	50	88.3
2.0	75	97.4
2.0	100	99.4

Table 3: Results of the power analysis, summarizing the power values determined for different assumptions about the general FBR effect and for different sample sizes. We ran a simulation with 5000 iterations for each parameter combination.

References

1. Meier R, Chmiel C, Valeri F, Muheim L, Senn O, Rosemann T. Long-Term Effects of Financial Incentives for General Practitioners on Quality Indicators in the Treatment of Patients With Diabetes Mellitus in Primary Care-A Follow-Up Analysis of a Cluster Randomized Parallel Controlled Trial. Front Med (Lausanne). 2021;8:664510. Epub 20211026. doi: 10.3389/fmed.2021.664510. PubMed PMID: 34765612; PubMed Central PMCID: PMC8576070.

2. R Core Team. R: A Language and Environment for Statistical Computing. R Foundation for Statistical Computing, Vienna, Austria Vienna, Austria2023. Available from: https://www.R-project.org/.

3. Bates D, Mächler M, Bolker BM, Walker SC. Fitting Linear Mixed-Effects Models Using lme4. Journal of Statistical Software. 2015;67(1):1-48. doi: 10.18637/jss.v067.i01.
